# Revolutionizing Chinese medicine granule placebo with a machine learning four-color model

**DOI:** 10.1186/s13020-024-01055-0

**Published:** 2025-04-01

**Authors:** Tingting Teng, Jingze Zhang, Peiqi Miao, Lipeng Liang, Xinbo Song, Dailin Liu, Junhua Zhang

**Affiliations:** 1 National Key Laboratory of Modern Chinese Medicine Innovation and Manufacturing, Tianjin University of Traditional Chinese Medicine, Tianjin, 301617 China; 2 Haihe Laboratory of Modern Chinese Medicine, Tianjin, 301617 China; 3 Tianjin Modern Innovation Chinese Medicine Technology Co., Ltd, Tianjin, 300380 China

**Keywords:** Traditional Chinese medicines, Placebo, Machine learning, Color prediction, Color similarity

## Abstract

With the development of new Chinese medicines and the need for clinical double-blind experiments, the use of placebos in Chinese medicine is becoming increasingly important. However, due to the diverse colors and complex color gamut of these particles, existing simulation methods rely on manual comparison and color mixing, leading to high subjectivity and errors. This study addresses this issue by developing a prediction model to accurately simulate the colors of Chinese medicine granules. In this study, 52 commercially available herbal particles were collected. And more than 320 simulated granules were prepared using fillers and four pigments (lemon yellow, carmine, indigo and caramel colors). Their RGB colors were collected using visible light imaging. A granule color prediction model was constructed by machine learning. First, the best clustering model was obtained by optimising the k-value of the Kmeans model. Subsequently, multiple regression models, including Gradient Boosting Regression (GBR), Support Vector Regression (SVR), and Random Forest, were evaluated through network search and cross-validation methods. Among these models, the average R^2^ of the random forest model reached 0.9249, outperforming other models. The prediction model accurately simulated the color of Chinese medicine granules, with an average color difference (ΔE) of 2.7734 and a high RGB value cosine similarity of 0.9999, alongside a 0.9366 similarity in artificial color scoring. This study introduces an innovative approach for the rapid and accurate prediction of granule colors, facilitating the development of clinically applicable placebos in traditional Chinese medicine.

**Supplementary Information:**

The online version contains supplementary material available at 10.1186/s13020-024-01055-0.

## Introduction

Traditional Chinese medicine (TCM) has played a significant role in healthcare across Asia and globally for thousands of years due to its distinctive therapeutic approaches. Countries like Japan and Korea have also embraced TCM practices, particularly the use of herbal granules [1]. Modern medicine has increasingly acknowledged TCM’s unique pharmacological effects and therapeutic potential [2, 3]. As TCM evolves and demand for clinical trials grows, the need for standardized methods to evaluate its effectiveness has become essential [4]. Placebos, which replicate the appearance and taste of active drugs but lack therapeutic ingredients, are critical for ensuring the integrity of these trials [5]. The similarity between placebos and active medicines directly influences the validity of trials, impacting their reliability and outcomes.

Achieving a close color match between herbal granules and placebos is vital for maintaining study blinding in clinical trials involving TCM. The colors of TCM granules are highly diverse, ranging from light yellowish-white to brownish yellow, reddish-brown, and dark black-brown. Subtle variations within these shades make precise color matching difficult. TCM granules need to be finely toned to ensure that they do not break blind in clinical trials due to colour differences. Conventionally, red, yellow, and blue are the primary colors used for blending to achieve specific hues. However, for TCM granules, the color range formed by the three primary colors is too wide, making it difficult to finely adjust the same color system. While this approach works for most applications, TCM granules primarily derive their colors from natural plant pigments, which typically exhibit brownish tones. The variations in these brownish tones are subtle and contrast sharply with the white base tones of the placebo excipients [6]. Given that most of the granules are brown, using a base color similar to brown proves to be a more scientific and effective choice. To address this discrepancy and refine the brown hues, incorporating specific brown pigments, in addition to red, yellow, and blue, is a more effective way to closely match the color of the herbal granules.

Existing color-matching methods have notable limitations. Typically, experienced personnel estimate an initial placebo color and refine it iteratively to achieve an acceptable match. However, this manual process is time-consuming, labor-intensive, and prone to human error and bias. Variability among personnel often necessitates multiple formulation attempts, leading to resource inefficiencies. Traditional methods also lack the precision and reproducibility required for large-scale clinical trials [7, 8]. Furthermore, the subjective nature of color perception exacerbates the challenge. Differences in individual perception can result in inconsistent matches, potentially influencing participants' expectations and subjective evaluations of the treatment's efficacy [9, 10]. This creates a risk of placebo effects that may either mask or amplify the actual therapeutic effects of the TCM being studied, thus undermining the validity of the clinical trial.

**Fig. 1:**
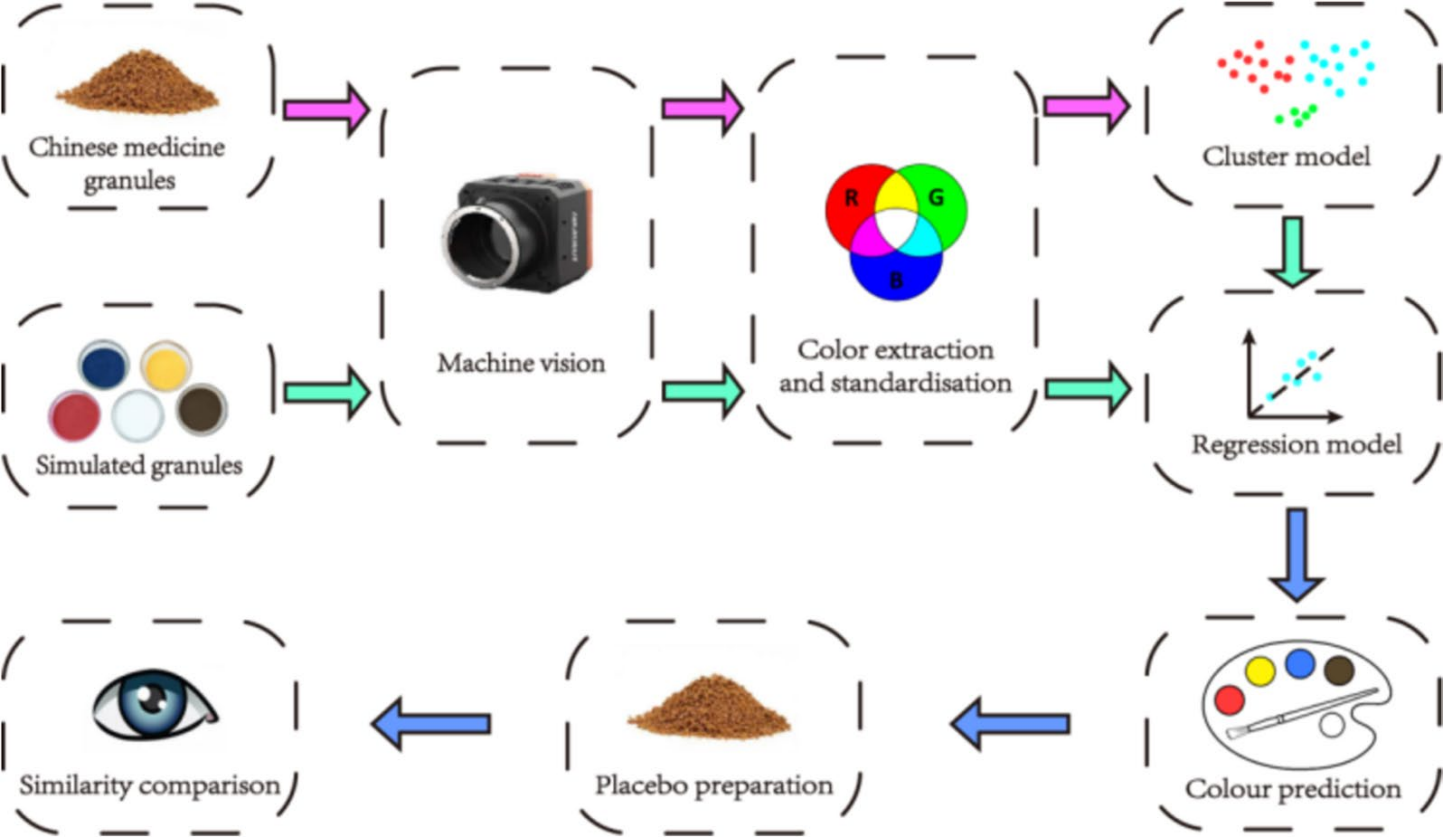
Chinese medicine granule placebo color prediction and similarity assessment process

TCM granule placebos have the limitation of being difficult to color precisely, with a high degree of subjectivity involved. To overcome these challenges, researchers have increasingly adopted visible light imaging and mathematical modeling to simulate TCM granule colors [11]. This study introduces a novel four-color simulation system incorporating red, yellow, blue, and brown pigments to achieve greater accuracy in matching TCM granule colors. By capturing color data through visible light imaging and training machine learning models on extensive datasets, this system addresses the limitations of traditional methods. Specifically, clustering models classify granule colors, while regression models predict corresponding placebo color schemes. These models are integrated into an automated system to select the most accurate color prediction model, enhancing the precision and reliability of placebo color matching. The system’s performance is validated through both machine vision and manual evaluation, providing a robust and reproducible approach to placebo preparation in double-blind trials (see Fig. 1).

## Materials and methods

### Sample preparation

Fifty-two commercially available herbal granules (Appendix 1) were collected, and 323 simulated granules were prepared using excipients and pigments to explore the relationship between pigments and color. The materials are detailed in Appendix 2. These materials are additives that can be used in accordance with Chinese national standards, and the amount of additives is within limits to ensure safety. Their solubility and stability were verified by pre-experiments to be in accordance with the experimental requirements. Excipients comprising lactose and dextrin in a 4:1 ratio were mixed with pigments (caramel, lemon yellow, carmine, and indigo) and adjusted with water to form granules. The granules were then ground into powder and sieved through a 180 μm ± 7.6 μm mesh to ensure uniformity.

### Data acquisition

The herbal and simulated granule powders were placed in round containers (30 mm diameter, 10 mm height), scraped flat, and photographed using a Hikvision industrial line scan camera equipped with an MV-LELS-300-2G-PY light source, MVL-LF5040M-F lens, and MV-CL042-90GC camera. Imaging parameters included an 18 μs exposure time, RGB 8 pixel format, 4096 × 5000 image resolution, 1380 Hz line frequency, and gamma correction set to 0.7000.

Using the Yolov5 target detection algorithm, a recognition model was developed to identify the target region (ROI), enabling rapid and accurate container location. The average RGB values of powders within the container were extracted using OpenCV image processing algorithms.

### Statistical analysis of data

RGB color planer and 3D scatter plots were generated to visualize the color distribution of herbal and simulated granules. Principal component analysis (PCA) was conducted using SIMCA 14.1 to reduce dimensionality and facilitate clustering visualization. Scatterplots provided a comprehensive overview of color patterns, aiding model development.

### Data preprocessing

The color data were standardized using the StandardScaler from Python's sklearn library (v3.11). Standardization involved calculating the mean and standard deviation for each feature and normalizing the data by subtracting the mean and dividing by the standard deviation. This ensured consistency in scaling between training and new data.

### Clustering model development

To cluster the herbal granules' color dataset, we employed the KMeans algorithm and evaluated the clustering performance using the sum of squared errors (SSE) and the silhouette coefficient [12]. SSE measures the degree of cohesion within a cluster by calculating the sum of squared distances between data points and their corresponding cluster centroids. The formula is as follows:

1 $$SSE = \sum\limits_{i = 1}^{m} {\left[ {dist\left( {x_{i} ,u_{i} } \right)} \right]^{{2}} }$$

where m represents the number of clusters, x_i_ denotes the data points within a cluster, and u_i_ is the centroid of that cluster.

To identify the optimal cluster number, we explored values ranging from 2 to 11 and plotted SSE against the number of clusters on an elbow plot. The "elbow" point, where the SSE curve starts to plateau, indicates the balance between minimizing within-cluster variance and avoiding excessive complexity [13, 14].

Subsequently, we computed the silhouette coefficient for each clustering result to further validate clustering quality. The silhouette coefficient evaluates clustering by comparing intra-cluster cohesion (average distance within a cluster) to inter-cluster separation (average distance to the nearest neighboring cluster). The formula is:

2 $$S = \frac{b - a}{{\max \left( {a,b} \right)}}$$

where: a is the average distance between a data point and all other points in the same cluster. b is the average distance between a data point and all points in the nearest neighboring cluster. Silhouette values range from −1 to 1, with higher values indicating better-defined clusters. We determined the optimal number of clusters by jointly analyzing the “elbow” point from the SSE plot and the silhouette coefficient's peak value [15].

The optimal cluster number was used to build a clustering model for the herbal granule dataset. This model was then applied to cluster the simulated granule colors, and results were stored in a "Cluster" column for further analysis.

### Regression model development

Regression predictions for each cluster of simulated granules were performed using grid search and cross-validation [16, 17]. RGB color data served as the independent variable, while the amount of pigment added was treated as the dependent variable. Several regression models, including Linear Regression, Random Forest, Gradient Boosting, Support Vector Regression, and Ridge Regression, were evaluated using Repeated K-Fold cross-validation with 5 folds. In each iteration, the model was trained on 4 folds and validated on onefold. The model with the best average Root Mean Square Error (RMSE) and R^2^ was selected for each cluster.

3 $$R^{2} = 1 - \frac{{\sum\limits_{i = 1}^{m} {\left( {y - \hat{y}} \right)^{2} } }}{{\sum\limits_{i = 1}^{m} {\left( {y - \overline{y}} \right)^{2} } }}$$

4 $$RMSE = \sqrt {\frac{{1}}{m}\sum\limits_{i = 1}^{m} {\left( {y - \hat{y}} \right)^{2} } }$$

To assess the importance of each feature in the model's predictions, we applied SHAP analysis. SHAP values were calculated to measure both global and local contributions, helping us understand how each feature influenced individual predictions [18]. This analysis provided valuable insights into the relative importance of the different features in the regression model.

**Fig. 2:**
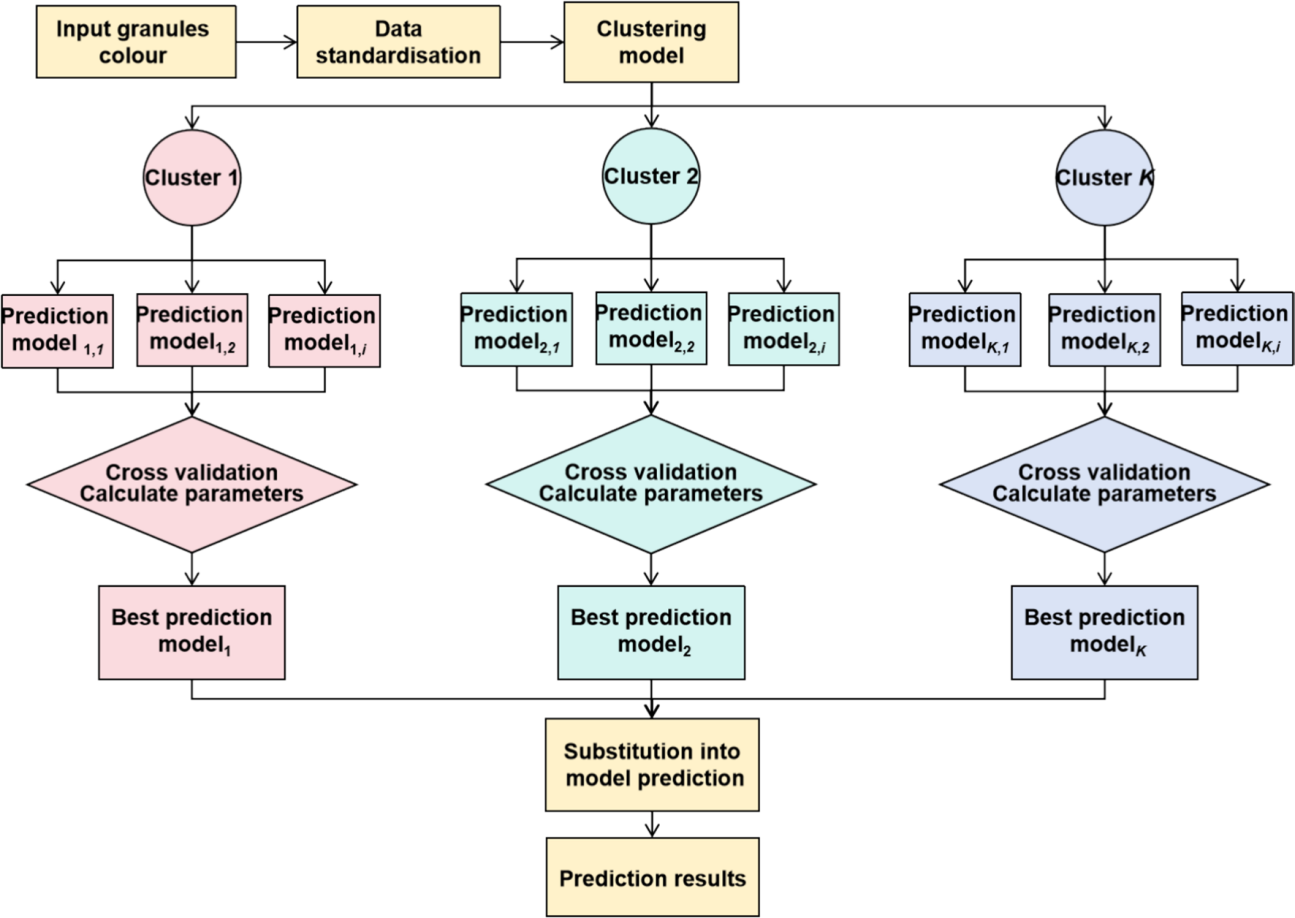
Prediction model for color matching of TCM granule placebo

The overall process for developing the TCM granule placebo color model is summarized in Fig. 2. First, the color data of the granules to be predicted is inputted and normalized. This normalized data is then passed into the clustering model, where the granules are assigned to corresponding clusters. Each cluster is associated with multiple regression models, and the best model is selected through cross-validation of the calculated parameters. Finally, the best regression model for each cluster is used to predict the placebo color, producing the color matching results.

### Model validation and evaluation

The color data of new TCM granules were input into the clustering model. Based on the predicted clusters, the appropriate regression model was applied to determine the placebo pigments, and the placebo granules were prepared accordingly. RGB values were converted to Lab values for a more accurate comparison, allowing us to calculate the color difference (ΔE) between the predicted and placebo granules. While RGB is suitable for color reproduction on monitors and lighting, Lab color space is ideal for comprehensive color management and precise color difference evaluations. ΔE is a commonly used method for comparing color differences, and its formula is:

5 $$\Delta E = \sqrt {\left( {L - L_{0} } \right)^{2} + \left( {a - a_{0} } \right)^{2} + \left( {b - b{}_{0}} \right)^{2} }$$

where L_0_, a_0_, and b_0_ represent the L, a, and b values of the freshness indicators in the initial state.

Since there is no uniform color difference standard for TCM placebos, we referred to the standards used in other fields, such as textiles and dyeing. The color difference thresholds are as follows: ΔE < 1 (nearly undetectable color difference), 1 ≤ ΔE < 3 (very small difference, generally not noticeable to the naked eye), 3 < ΔE < 6 (slightly perceptible differences), 6 < ΔE < 9 (fairly perceptible differences), 9 < ΔE < 12 (clearly perceptible differences), and ΔE > 12 (different colors) [19–21].

Additionally, the cosine (COS) value, computed from RGB values, reflects the color similarity between the placebo and TCM granules. A COS value closer to 1 indicates higher similarity [22, 23]. By analyzing both ΔE and COS results, we provide a comprehensive assessment of the predictive model’s performance and reliability.

6 $$COS\left( \theta \right) = \frac{a \cdot b}{{\left\| a \right\| \cdot \left\| b \right\|}}$$

To further evaluate the model, single-blind randomization principles were applied. TCM granules served as the reference, and both the TCM granules and their placebos were randomly divided into Drug A and Drug B groups. Twenty adults with normal color vision assessed the similarity between the drugs using the following scoring system: 0 points (completely inconsistent), 1 point (slightly close), 2 points (moderately close), and 3 points (identical).

Statistical analysis was performed using SPSS 21.0 software, employing paired t-tests with an α = 0.05 significance level to process the scoring data (X ± S). Both machine-generated and manual evaluations were combined to validate and assess the integrated simulation of the model.

## Results and discussion

### Statistical analysis of data

**Fig. 3:**
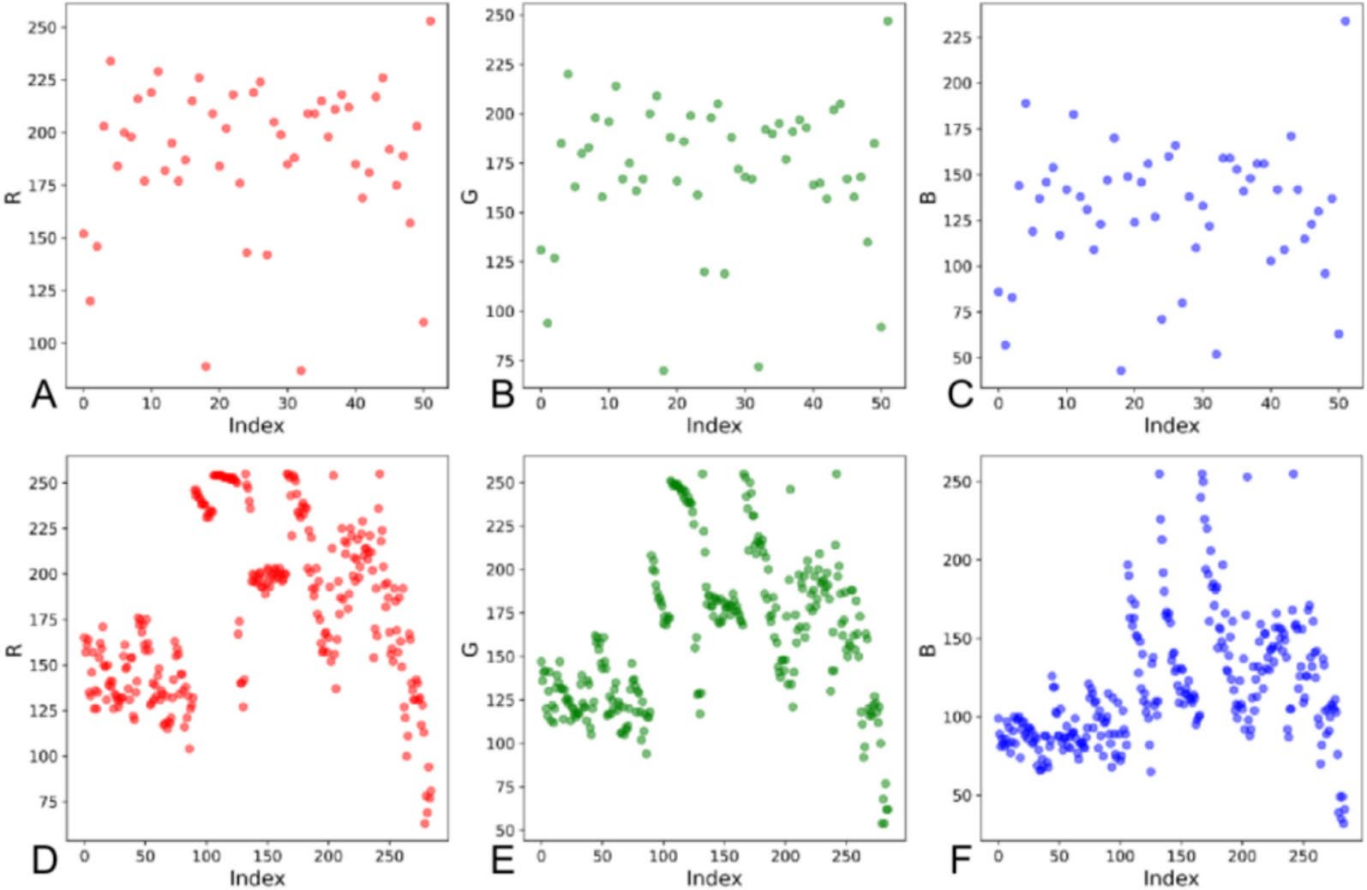
Granules RGB color scatterplot: **A** R-value color planar scatterplot of TCM granules, **B** G-value color planar scatterplot of TCM granules, **C** B-value color planar scatterplot of TCM granules, **D** R-value color planar scatterplot of simulated granules, **E** G-value color planar scatterplot of simulated granules, **F** B-value color planar scatterplot of simulated granules

The TCM granules exhibited a color range of R: 87–253, G: 70–247, and B: 43–234, while the simulated granules ranged from R: 63–255, G: 54–255, and B: 32–255. RGB color scatter plots for both granule types (Fig. 3) reveal a scattered and disordered distribution of color data. Given this complexity, a single regression model struggles to capture the subtle color variations. To address this, we applied cluster analysis to segment the color data into distinct groups, each with its own cluster center. Regression analysis was then performed for each cluster, creating a hierarchical prediction model that enhances both accuracy and precision by more effectively capturing the color trends of the granules.

**Fig. 4:**
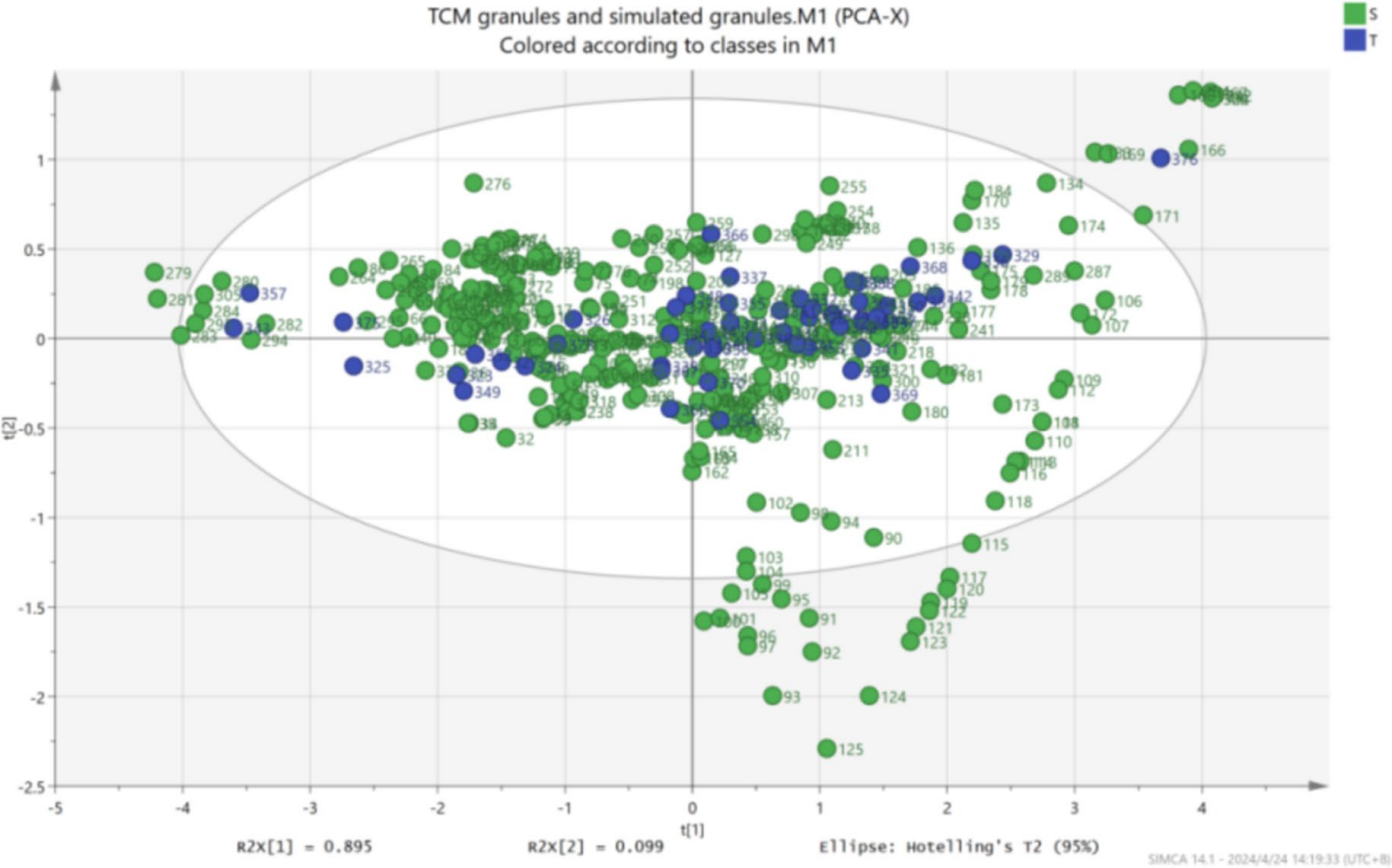
PCA analysis of TCM granules and simulated granules

PCA analysis of the color data (Fig. 4) reveals that the color range of TCM granules is largely encompassed within the range of the simulated granules, with T representing TCM granules and S representing simulated granules. This finding demonstrates that the predictive regression model using simulated granule colors adequately covers the color range of TCM granules, enabling a comprehensive simulation.

### Clustering model performance

**Fig. 5:**
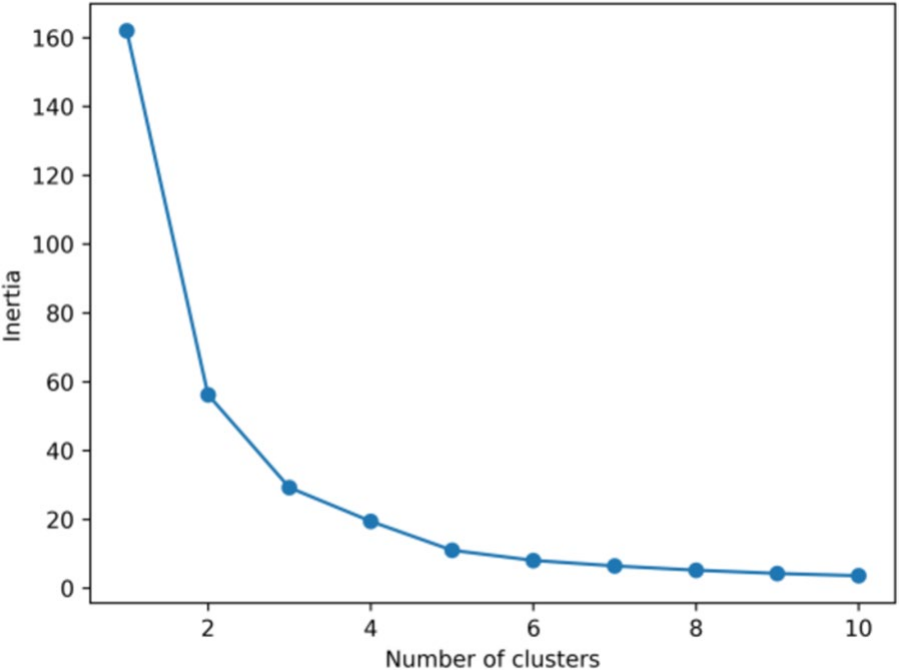
Elbow plot of KMeans clustering for TCM granules

**Fig. 6:**
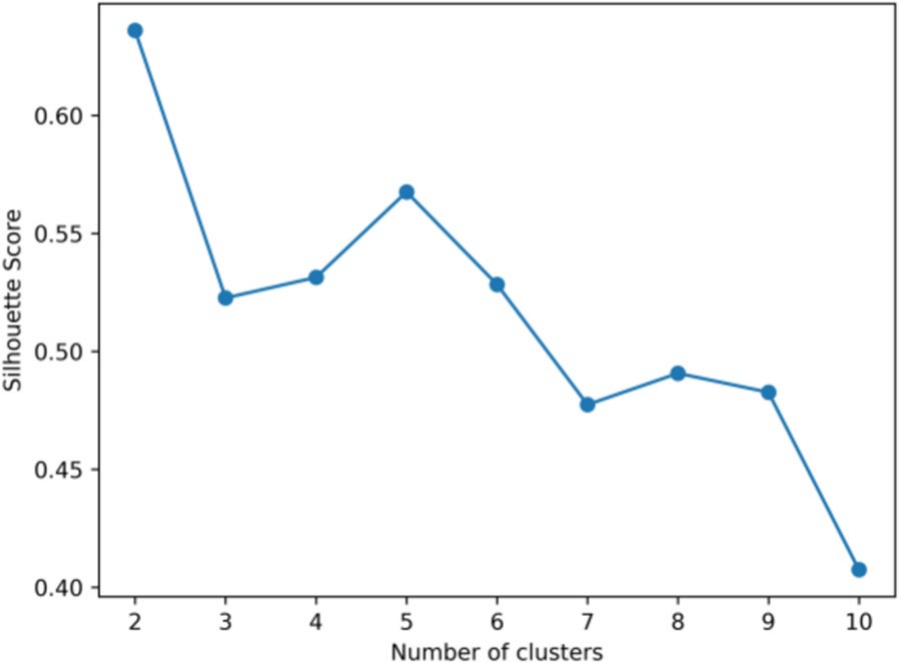
Silhouette coefficients of KMeans clustering for TCM granules

**Fig. 7:**
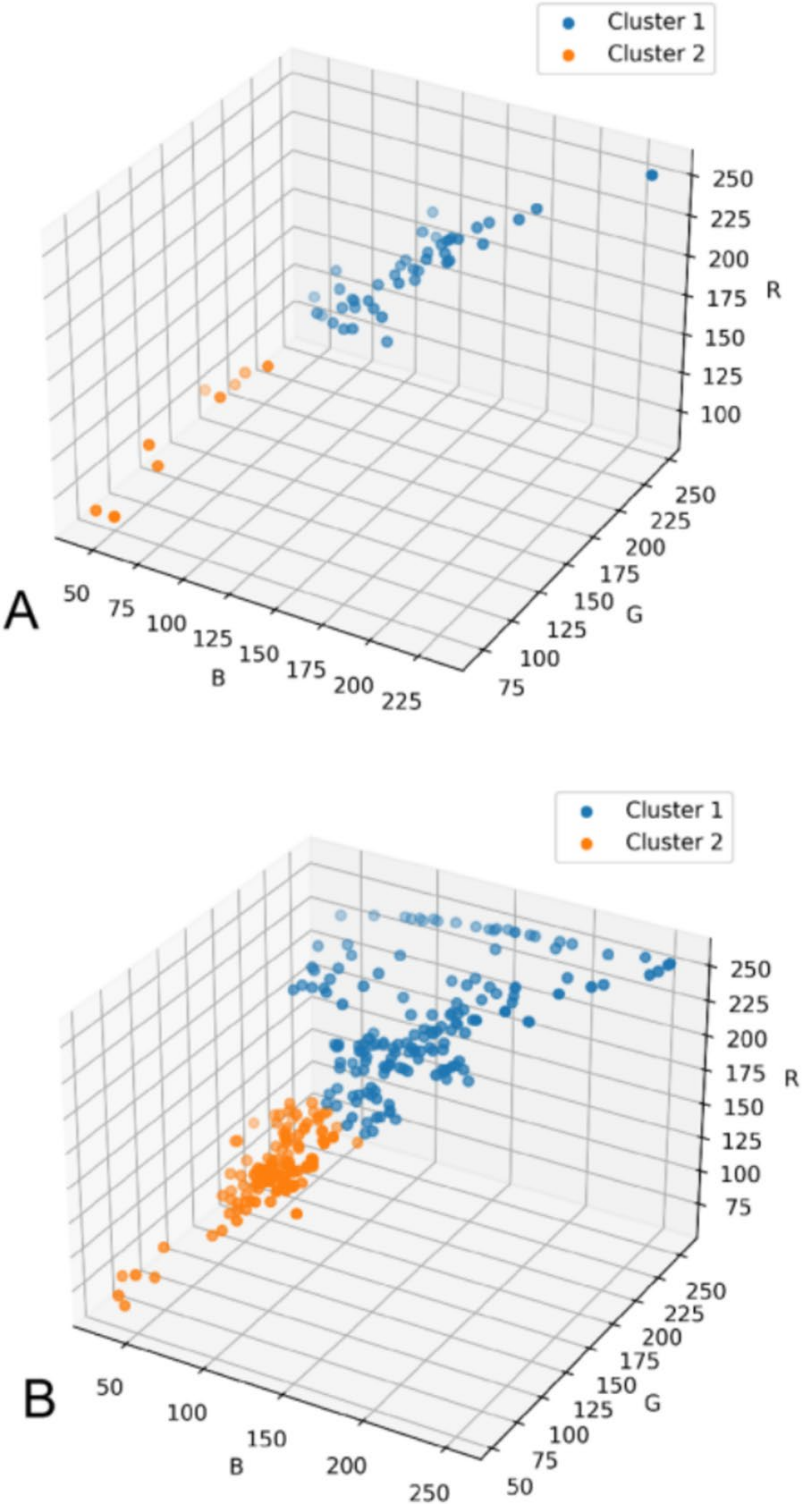
RGB color scatter plot after clustering: (A) 3D scatter plot of RGB color clustering of TCM granules, (B) 3D scatter plot of RGB color clustering of simulated granules

The KMeans algorithm clustered the TCM granule color dataset into 2 to 11 groups. The elbow plot (Fig. 5) revealed a significant reduction in within-cluster variance starting at 2 clusters, indicating an improvement in clustering quality. Although the elbow plot provides guidance on cluster selection, additional metrics, such as the silhouette coefficient, are necessary for further validation. The silhouette plot (Fig. 6) indicated the highest coefficient of 0.6360 at k = 2, suggesting that two clusters offer the best clustering quality. This reflects distinct color distributions and supports that k = 2 is the optimal choice for classifying TCM granule colors. The 3D scatter plot (Fig. 7A) confirms this, clearly showing the separation of the granules into two distinct color categories, which may highlight key attributes of the TCM granule colors.

Next, the simulated granule colors were input into the saved KMeans model, which assigned them to the appropriate centroids. The 3D scatter plot (Fig. 7B) demonstrates that the simulated granules were divided into two groups, facilitating further regression modeling.

### Regression model performance

Regression predictions for the simulated granules used RGB color as the independent variable and pigment amount as the dependent variable. Several regression models were evaluated, with optimal parameters determined through Repeated K-Fold cross-validation and grid search. The correlation coefficients and performance statistics are provided in Table 1. The Random Forest regression model achieved correlation coefficients of 0.9305 and 0.9193 for the two clusters, with an average R^2^ of 0.9249 and mean square errors of 20.6466 and 3.1318, respectively. These results indicate that the Random Forest model outperforms the other models.

**Table 1:** Regression Model performance comparison

Cluster	Regression Model	Average RMSE	Average R^2^
1	LinearRegression	56.5449	0.4618
1	RandomForestRegressor	20.6466	0.9305
1	GradientBoostingRegressor	30.7755	0.8382
1	SVR	78.7574	0.2451
1	Ridge	56.5589	0.4614
2	LinearRegression	8.1846	0.5192
2	RandomForestRegressor	3.1318	0.9193
2	GradientBoostingRegressor	5.3877	0.8169
2	SVR	7.2599	0.6270
2	Ridge	8.1847	0.5192

**Fig. 8:**
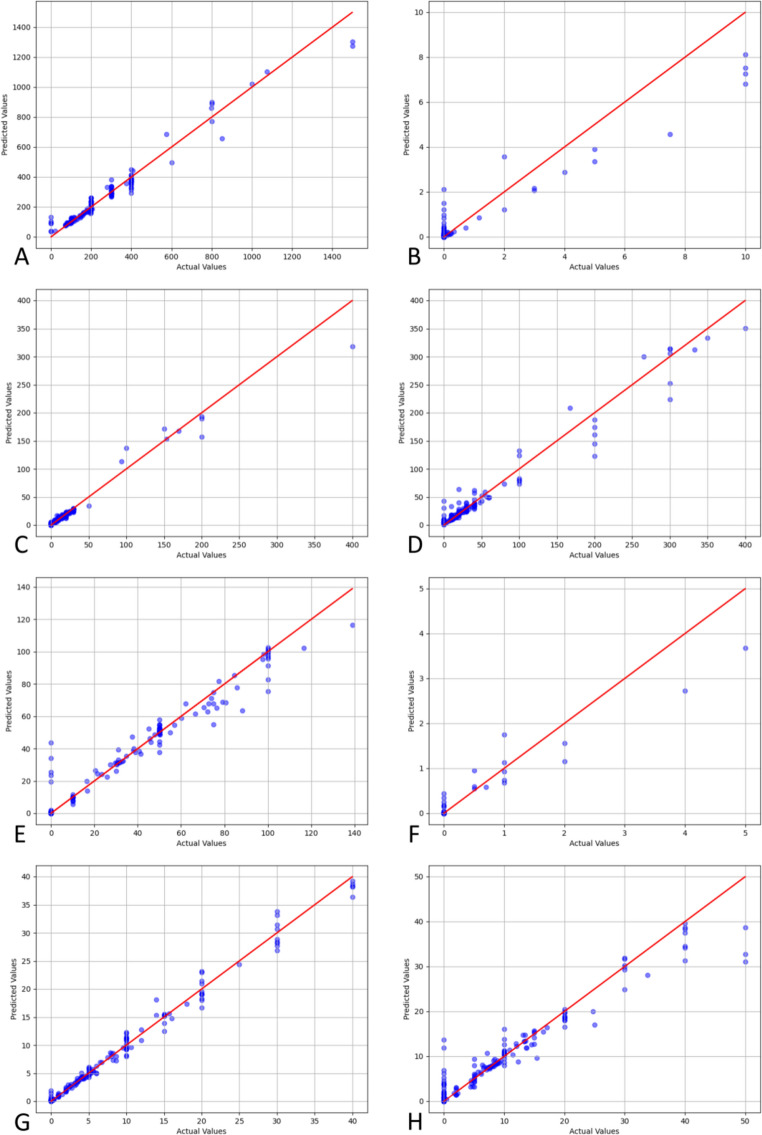
Correlation between predicted and actual pigment addition values using the Random Forest Regressor: **A** Caramel color addition for cluster 1, **B** Indigo addition for cluster 1, **C** Lemon yellow addition for cluster 1, **D** Carmine addition for cluster 1, **E** Caramel color addition for cluster 2, **F** Indigo addition for cluster 2, **G** Lemon yellow addition for cluster 2, **H** Carmine addition for cluster 2

The higher RMSE in Cluster 1 is attributed to the significant influence of caramel color, as all granules in this cluster are light-colored. This leads to greater variation in color as pigment amounts change. To compare actual and predicted values, we created a scatter plot with a reference diagonal line (Fig. 8). Most data points closely align with the diagonal, suggesting a strong linear relationship. However, deviations increase with larger pigment additions, likely due to the overall darkening of the granules' color, which can obscure the color representation.

**Fig. 9:**
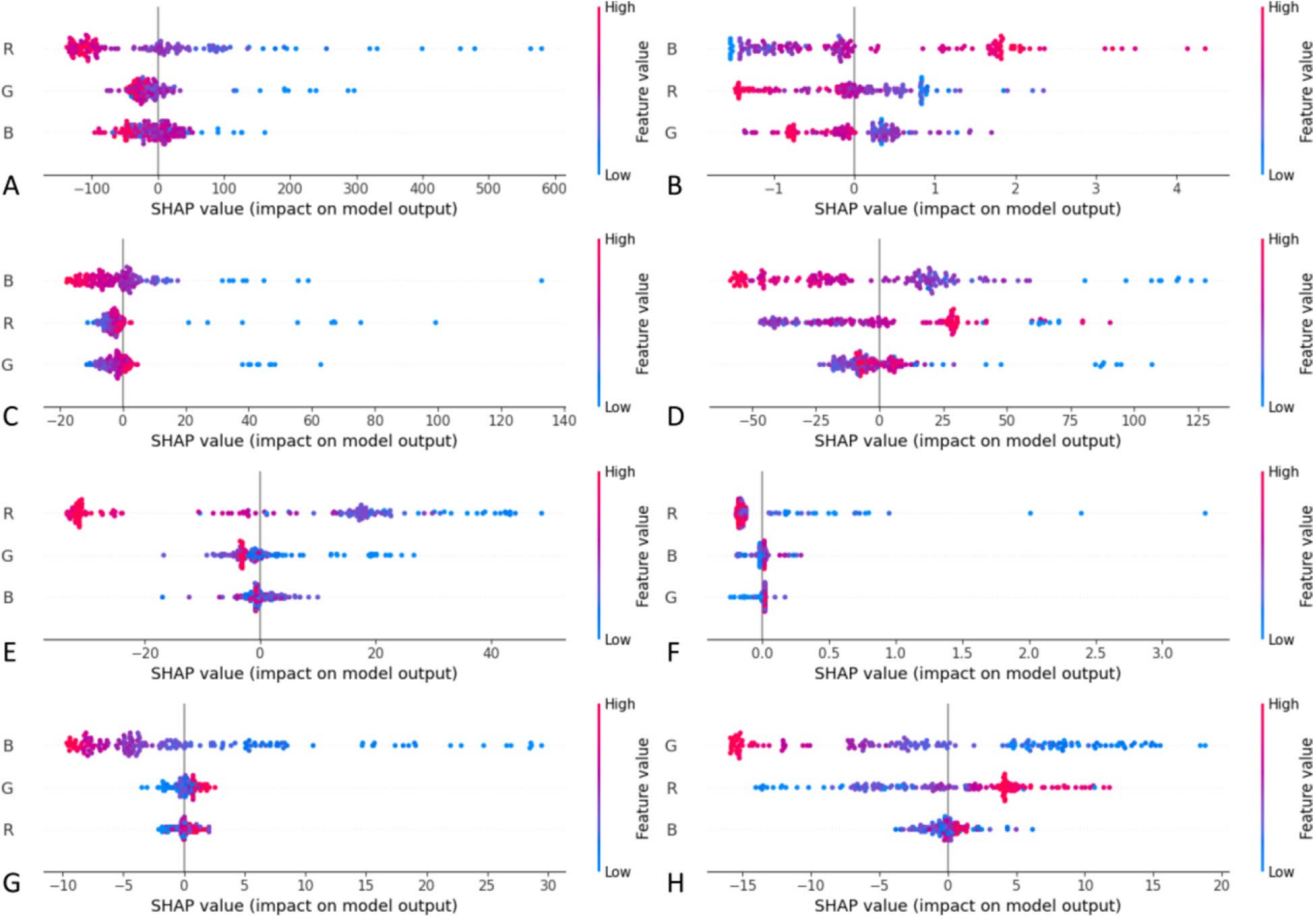
Distribution of SHAP values for the random forest model: **A** Caramel color SHAP overall impact plot in cluster 1, **B** Indigo SHAP overall impact plot in cluster 1, **C** Lemon yellow SHAP overall impact plot in cluster 1, **D** Carmine SHAP overall impact plot in cluster 1, **E** Caramel color SHAP overall impact plot in cluster 2, **F** Indigo SHAP overall impact plot in cluster 2, **G** Lemon yellow SHAP overall impact plot in cluster 2, **H** Carmine SHAP overall impact plot in cluster 2

The SHAP analysis of the best-performing model provided valuable insights into the contributions of each feature in predicting pigment addition amounts. Figure 9 illustrates the SHAP values for individual samples, showing both the direction and magnitude of each feature's impact on the predictions. In Cluster 1, the prediction for caramel color was primarily influenced by the R (red) value, while lemon yellow and indigo were mainly influenced by the B (blue) value, and the prediction for carmine was most affected by the G (green) value. In Cluster 2, caramel and indigo predictions were dominated by the R value, lemon yellow was influenced by the B value, and carmine was most impacted by the G value.

The caramel color, which ranges from a brown-black hue to a lighter shade, darkens as more pigment is added, leading to lower RGB values. Consequently, the RGB values decrease with increasing caramel pigment, with the R value being the most significant factor. To the naked eye, caramel appears as dark brown to russet. From a color theory perspective, red is typically the dominant component in brown hues, which aligns with the findings from the SHAP analysis. For lemon yellow, the B value inversely affects the amount added due to the hue's opposition to blue-purple. Similarly, the G value has the most significant inverse effect on the addition of carmine, while the R value has a positive effect. The B value significantly and positively influences the addition of indigo. In Cluster 2, with lighter particles and minimal indigo addition, the R value inversely affects indigo addition. This is because light-colored granules are almost always light yellow or light reddish brown, and light blue granules are nearly non-existent. In the hue ring, the distance between blue and red is much greater than between blue and green. When blue is not the dominant color, red has the strongest negative influence on the amount of indigo added.

In conclusion, we analyzed the regression model by identifying the minimum RMSE and maximum R^2^ values, plotting actual versus predicted values, and performing SHAP analysis. These steps allowed us to identify the optimal predictive model, evaluate its performance, and understand the contributions of each feature to the predictions.

### External validation of color predictions

**Fig. 10:**
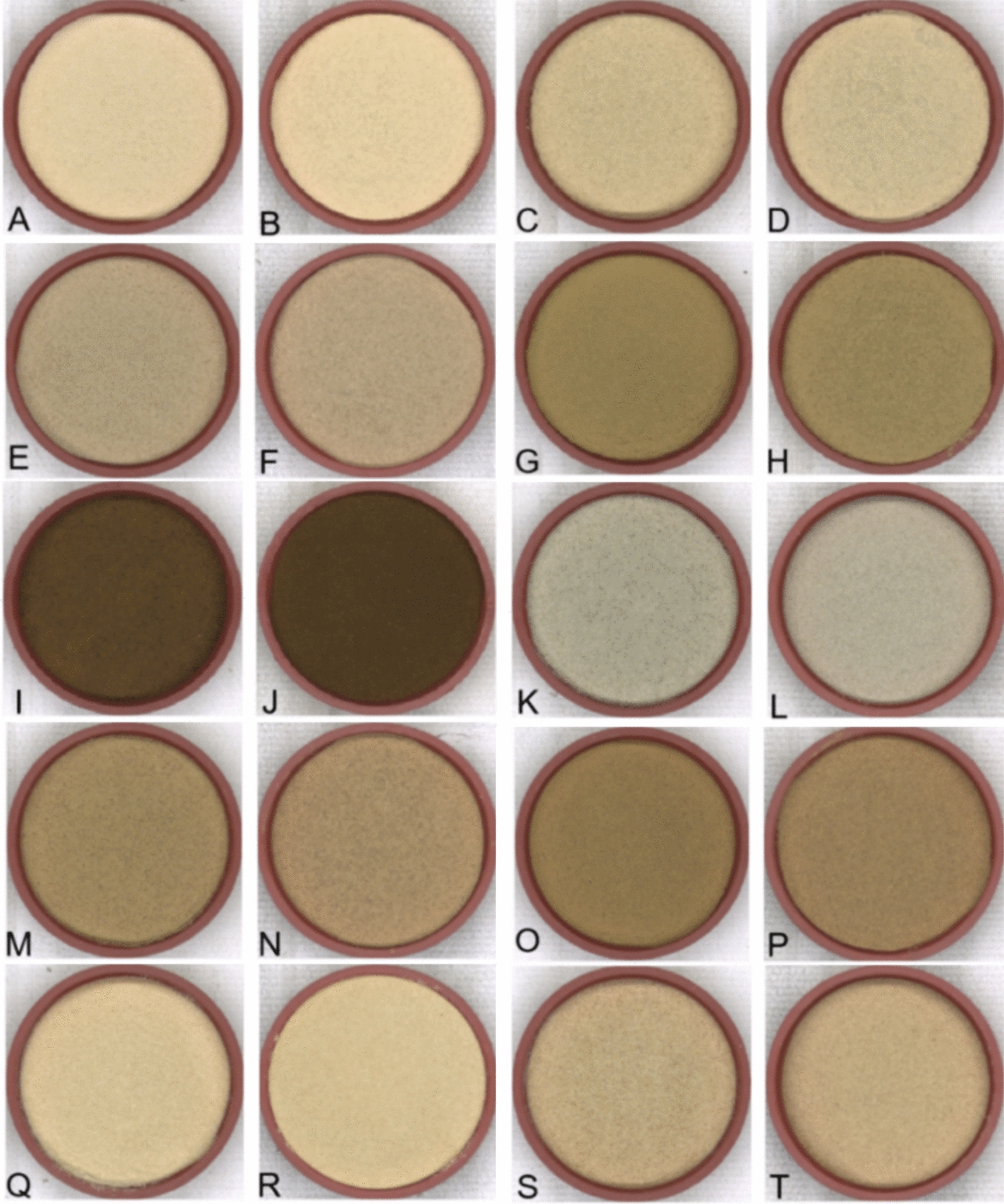
TCM granules and theirs placebo granules: **A** No.1 TCM granules, **B** No.1 placebo granules, **C** No.2 TCM granules, **D** No.2 placebo granules, **E** No.3 TCM granules, **F** No.3 placebo granules, **G** No.4 TCM granules, **H** No.4 placebo granules, **I** No.5 TCM granules, **J** No.5 placebo granules, **K** No.6 TCM granules, **L** No.6 placebo granules, **M** No.7 TCM granules, **N** No.7 placebo granules, **O** No.8 TCM granules, **P** No.8 placebo granules, **Q** No.9 TCM granules, **R** No.9 placebo granules, **S** No.10 TCM granules, **T** No.10 placebo granules

Ten herbal granules were predicted and granulated, with the results shown in Table 2. A preliminary comparison between the TCM granules and their corresponding placebos is illustrated in Fig. 10. The color difference (ΔE) and cosine similarity (COS) values between the predicted and placebo granules were calculated, with the specific results provided in Table 3. All ten granules had ΔE values less than 6, and six granules had values less than or equal to 3, indicating minimal perceptible differences. The COS values, where a value closer to 1 indicates higher similarity, showed that the placebos were highly similar to the original granules. The average ΔE was 2.7734, and the average RGB similarity was 0.9999, demonstrating strong performance in color prediction. Manual sensory evaluations also showed high color similarity, with an overall similarity score of 0.9366, as detailed in Table 4. Additionally we verified model stability and the absence of overfitting in the supplementary document.

**Table 2:** Results of color matching prediction for TCM granules placebo

Name	Caramel color(mg)	Lemon yellow (mg)	Carmine (mg)	Indigo (mg)	Excipient (g)
No.1 placebo	25.79	3.19	7.22	0.00	20.00
No.2 placebo	45.14	4.56	8.41	0.00	20.00
No.3 placebo	91.85	3.81	15.36	0.00	20.00
No.4 placebo	181.83	20.31	25.11	0.05	20.00
No.5 placebo	797.40	153.02	265.12	0.00	20.00
No.6 placebo	85.53	0.16	3.25	0.73	20.00
No.7 placebo	155.01	13.7	48.37	0.00	20.00
No.8 placebo	181.84	17.42	56.61	0.00	20.00
No.9 placebo	34.74	3.55	7.94	0.00	20.00
No.10 placebo	70.5	5.53	17.11	0.02	20.00

**Table 3:** Color data and similarity of TCM granules and their placebo

Name	B	G	R	L	a	b	ΔE	COS
No.1 TCM granules	166	205	224	83	1	22	2.4495	1.0000
No.1 placebo	166	209	229	84	0	24		
No.2 TCM granules	144	185	203	76	0	23	2.0000	0.9999
No.2 placebo	148	186	203	76	0	21		
No.3 TCM granules	127	159	176	66	1	19	1.4142	1.0000
No.3 placebo	129	161	181	67	2	19		
No.4 TCM granules	83	127	146	54	1	27	3.6056	0.9998
No.4 placebo	92	134	152	57	1	25		
No.5 TCM granules	43	70	89	31	4	19	3.0000	0.9999
No.5 placebo	37	63	80	28	4	19		
No.6 TCM granules	142	165	169	68	−3	12	3.6056	1.0000
No.6 placebo	151	173	181	71	−1	12		
No.7 TCM granules	96	135	157	57	3	24	3.3166	0.9999
No.7 placebo	105	142	166	60	4	23		
No.8 TCM granules	80	119	142	51	3	25	5.1962	0.9998
No.8 placebo	92	130	154	56	4	24		
No.9 TCM granules	153	195	215	79	1	24	1.4142	1.0000
No.9 placebo	150	194	214	79	0	25		
No.10 TCM granules	130	168	189	70	2	22	1.7321	1.0000
No.10 placebo	132	171	195	71	3	23

**Table 4:** Results of manual evaluation of similarity between Chinese granules and their placebo colors (X ± S)

No	TCM granules	placebo granules	*P*	COS
No.1	2.20 ± 0.70	2.55 ± 0.60	0.069	0.9460
No.2	2.35 ± 0.59	2.10 ± 0.72	0.135	0.9526
No.3	2.45 ± 0.60	2.05 ± 0.69	0.017	0.9570
No.4	1.95 ± 0.89	2.10 ± 0.72	0.481	0.9258
No.5	2.10 ± 0.64	1.80 ± 0.70	0.110	0.9254
No.6	2.10 ± 0.85	2.30 ± 0.73	0.330	0.9285
No.7	1.95 ± 0.60	1.65 ± 0.75	0.083	0.9107
No.8	1.95 ± 0.76	1.70 ± 0.73	0.204	0.9101
No.9	2.35 ± 0.67	2.15 ± 0.59	0.258	0.9490
No.10	2.40 ± 0.60	2.10 ± 0.64	0.055	0.9610

In summary, we validated the predictive capability of our model by accurately forecasting the pigment amounts for TCM granules and using these predictions in the granulation process of the placebos. Our analysis of ΔE and COS values confirmed the model’s accuracy and reliability in predicting the color of the granules.

## Discussion

In this study, we developed a clustering-based regression model to simulate the colors of TCM placebo granules. A significant innovation was the introduction of a four-color simulation system—red, yellow, blue, and brown—which expanded the color range and improved the precision of matching TCM granule colors. To ensure uniformity, the granules were ground into powder for color collection. This approach is not limited to TCM granules but can also be extended to other solid preparations, such as various types of granules, powders, and tablets. Any medication that can be converted into a powdered form is suitable for this simulation method.

While RGB color space is widely used in digital imaging for its compatibility with image sensors and display devices, it only captures three color channels—red, green, and blue. To enhance the accuracy of the model, incorporating other color spaces such as HSV (hue, saturation, value) and CMYK (cyan, magenta, yellow, black) could provide additional dimensions of color information [24]. HSV, particularly useful for adjusting brightness and darkness, can improve accuracy in certain scenarios [25]. CMYK, commonly used in painting and printing, better reflects the effects of pigment mixing and can enhance color reproduction for specific formulations [26, 27]. While this study primarily relied on the RGB color space for its intuitive representation of light intensity, future work will incorporate HSV and CMYK color spaces to improve accuracy and practicality.

To improve adaptability, factors such as device differences, viewing angles, and light sources should also be considered. These factors can affect color perception and, consequently, the model’s generalization and robustness. In this study, a whiteboard with approximately 100% reflectance was used for correction, adjusting the RGB value of the whiteboard to 255. Future studies will evaluate the model’s performance under varying lighting conditions, such as natural and artificial light sources. Parameters like color temperature, illumination intensity, and light source uniformity will be examined to ensure the model's stability in practical applications.

The predictive model’s database should be continuously expanded and updated. As more placebo formulations are developed, incorporating new color-matching data will enhance the model’s predictive capabilities to meet production requirements. In addition to the models used in this study, exploring deep learning and neural networks could further improve particle color prediction. Deep learning models, with their ability to automatically learn features, and neural networks, with their hierarchical structure and powerful fitting capabilities, are particularly effective in addressing complex color prediction challenges [28]. We plan to apply various deep learning algorithms for multidimensional color data. Algorithms such as DNN, CNN, Transformer, and Mamba can be used for feature extraction and optimization of model performance across RGB, HSV, CMYK, and other color spaces to reduce errors. DNNs could be explored for capturing complex patterns in color data, potentially improving feature extraction and prediction accuracy. CNNs might be used to investigate their capability to extract spatial features from color images, which could help address variations in particle shape and surface texture that influence color [29]. Transformers may be examined for their ability to model long-range dependencies in color data, especially under varying lighting conditions [30]. Additionally, we might explore Mamba networks for their reported efficiency in processing high-dimensional data, which could aid in generalizing across different pigment formulations [31]. We will also consider error reduction and performance optimization techniques, such as dropout, regularization, and data augmentation, to enhance the model's generalization capability for unseen data. Integrating these advanced methods is expected to drive significant advancements in the field.

To improve the accuracy of granule color similarity assessments, lowering the threshold for color difference values can allow for more precise distinctions. Incorporating color cards can further enhance the objectivity of manual scoring [7]. These cards provide standard color samples for direct comparison, reducing subjective bias and ensuring consistency and reliability in evaluations [32]. Thus, optimizing color difference thresholds and utilizing color cards in scoring methods are recommended when developing standards for granule color similarity.

## Conclusion

In this study, RGB colors of 52 commercially available Chinese medicine granules were collected, and simulated granules were prepared using fillers and four pigments. A color prediction model was developed by combining clustering and regression models through machine learning. Optimizing the k-value of the K-means model identified the best clustering model, and network search and cross-validation selected the best regression model. The random forest regression model, achieving an average R^2^ of 0.9249, was chosen for color prediction. The model predicted the color of ten TCM granules, with an average color difference of 2.7734 and an average RGB value similarity of 0.9999. Manual scoring showed a similarity of 0.9366, and randomized single-blind experiments confirmed a high degree of similarity.

This model effectively meets the requirements for large-scale color simulation of TCM granules, providing fast and accurate color fitting. Given the complexity of simulating TCM granule colors and the need for placebo controls in clinical trials, this predictive model is valuable for developing clinically viable placebos and advances the field of placebo color prediction.

## Supplementary Information

Supplementary material 1.

Supplementary material 2.

Supplementary material 3.

## Data Availability

Data will be made available on request, please contact the corresponding author.
