# Supplementary material for: Revolutionizing Chinese medicine granule placebo with a machine learning four-color model
Source: Chin Med. 2025 Apr 1;20:43. doi: 10.1186/s13020-024-01055-0 (PMC11963323; doi:10.1186/s13020-024-01055-0)
Supplement: Supplementary file 1 — Supplementary material 1. [file 13020_2024_1055_MOESM1_ESM.docx]

**Supplementary document**

**Table 1S**

Information of the 10 test set TCM granules.

| No. | TCM Granules | Manufacture company | Batch |
| --- | --- | --- | --- |
| 1 | Ganmaoling Granules | Yunnan Yufeng Pharmaceutical Co., Ltd | 2312088 |
| 2 | Xiao'er Kechuanling Granules | Sunflower Pharmaceutical Group (Xiangyang) Longzhong Co., Ltd | 221204 |
| 3 | Tianma Gouteng Granules | Chengdu Jiuzhitang Jinding Pharmaceutical Co., Ltd | 221202 |
| 4 | Yimucao granules | Sichuan Yike Pharmaceutical Co., Ltd | 241002 |
| 5 | Fenghan Ganmao Granules | Shaanxi Ziguang Chenji Pharmaceutical Co., Ltd | 824151 |
| 6 | Xiao'er Magan Granules | Sunflower Pharmaceutical Group Hubei Wudang Co., Ltd | 20240107 |
| 7 | Lvmei Zhixie Granules | Anhui Weimin Pharmaceutical Co., Ltd | 240509 |
| 8 | Xuanmai Ganju granules | Sichuan Yike Pharmaceutical Co., Ltd | 240905 |
| 9 | Xingsu Zhike Granules | Yili Pharmaceutical (Luoding) Co., Ltd | 240902 |
| 10 | Xiao'er Xiesuting Granules | Harbin Children's Pharmaceutical Factory Co., Ltd | 220601 |

Ten types of commercially available Chinese herbal granules, which were not included in the model's training set, were collected as the test set. The sources of the granules are listed in Table 1S.

**Table 2S**

Colour values of TCM granules.

| No. | B | G | R | B mean | G mean | R mean | B std | G std | R std | RSD |
| --- | --- | --- | --- | --- | --- | --- | --- | --- | --- | --- |
| 1-1 | 160 | 197 | 217 | 161 | 198 | 218 | 1.15 | 1.15 | 0.58 | 0.52% |
| 1-2 | 162 | 199 | 218 |  |  |  |  |  |  |  |
| 1-3 | 162 | 199 | 218 |  |  |  |  |  |  |  |
| 2-1 | 98 | 142 | 170 | 98 | 142 | 170 | 0.00 | 0.58 | 0.58 | 0.25% |
| 2-2 | 98 | 143 | 171 |  |  |  |  |  |  |  |
| 2-3 | 98 | 142 | 170 |  |  |  |  |  |  |  |
| 3-1 | 63 | 103 | 126 | 63 | 103 | 126 | 0.58 | 0.58 | 0.58 | 0.65% |
| 3-2 | 63 | 104 | 127 |  |  |  |  |  |  |  |
| 3-3 | 62 | 103 | 126 |  |  |  |  |  |  |  |
| 4-1 | 153 | 192 | 210 | 152 | 191 | 209 | 1.53 | 1.00 | 1.15 | 0.69% |
| 4-2 | 150 | 190 | 208 |  |  |  |  |  |  |  |
| 4-3 | 152 | 191 | 210 |  |  |  |  |  |  |  |
| 5-1 | 107 | 153 | 178 | 107 | 153 | 178 | 0.00 | 0.58 | 0.00 | 0.13% |
| 5-2 | 107 | 154 | 178 |  |  |  |  |  |  |  |
| 5-3 | 107 | 153 | 178 |  |  |  |  |  |  |  |
| 6-1 | 161 | 210 | 233 | 161 | 210 | 233 | 0.00 | 0.00 | 0.00 | 0.00% |
| 6-2 | 161 | 210 | 233 |  |  |  |  |  |  |  |
| 6-3 | 161 | 210 | 233 |  |  |  |  |  |  |  |
| 7-1 | 181 | 208 | 229 | 180 | 208 | 229 | 0.58 | 0.58 | 0.00 | 0.20% |
| 7-2 | 180 | 209 | 229 |  |  |  |  |  |  |  |
| 7-3 | 180 | 208 | 229 |  |  |  |  |  |  |  |
| 8-1 | 144 | 168 | 181 | 144 | 168 | 181 | 0.00 | 0.00 | 0.00 | 0.00% |
| 8-2 | 144 | 168 | 181 |  |  |  |  |  |  |  |
| 8-3 | 144 | 168 | 181 |  |  |  |  |  |  |  |
| 9-1 | 201 | 230 | 247 | 202 | 230 | 246 | 0.58 | 0.00 | 0.58 | 0.17% |
| 9-2 | 202 | 230 | 246 |  |  |  |  |  |  |  |
| 9-3 | 202 | 230 | 246 |  |  |  |  |  |  |  |
| 10-1 | 125 | 162 | 183 | 125 | 162 | 183 | 1.00 | 0.58 | 0.58 | 0.49% |
| 10-2 | 124 | 162 | 182 |  |  |  |  |  |  |  |
| 10-3 | 126 | 163 | 183 |  |  |  |  |  |  |  |

Three parallel image captures were repeated, and the average RGB values were calculated along with their standard deviations. The RSD values for the RGB values were then calculated and averaged to obtain the overall RSD. The results are shown in Table 2S. The RSD values were all below 2%, indicating that the sample collection was uniform and stable.

**Table 3S**

Results of color matching prediction for placebo granules.

| No. | Caramel color  (mg) | Lemon yellow  (mg) | Carmine  (mg) | Indigo  (mg) | Caramel  mean | Lemon yellow  mean | Carmine  mean | Indigo  mean | Caramel  std | Lemon yellow  std | Caramel  std | Indigo  std | RSD |
| --- | --- | --- | --- | --- | --- | --- | --- | --- | --- | --- | --- | --- | --- |
| 1-1 | 32.75 | 3.57 | 8.94 | 0.07 | 32.20 | 3.50 | 8.80 | 0.07 | 0.48 | 0.06 | 0.12 | 0.00 | 1.13% |
| 1-2 | 31.92 | 3.47 | 8.73 | 0.07 |  |  |  |  |  |  |  |  |  |
| 1-3 | 31.92 | 3.47 | 8.73 | 0.07 |  |  |  |  |  |  |  |  |  |
| 2-1 | 114.70 | 14.21 | 30.93 | 0.11 | 114.07 | 14.31 | 30.77 | 0.11 | 1.09 | 0.17 | 0.28 | 0.00 | 0.77% |
| 2-2 | 112.82 | 14.51 | 30.44 | 0.11 |  |  |  |  |  |  |  |  |  |
| 2-3 | 114.70 | 14.21 | 30.93 | 0.11 |  |  |  |  |  |  |  |  |  |
| 3-1 | 417.14 | 69.67 | 147.66 | 0.00 | 413.78 | 69.72 | 145.03 | 0.00 | 6.03 | 1.01 | 2.75 | 0.00 | 1.20% |
| 3-2 | 406.82 | 68.73 | 142.17 | 0.00 |  |  |  |  |  |  |  |  |  |
| 3-3 | 417.37 | 70.75 | 145.26 | 0.00 |  |  |  |  |  |  |  |  |  |
| 4-1 | 39.52 | 3.64 | 8.50 | 0.11 | 40.11 | 3.73 | 8.66 | 0.11 | 1.11 | 0.08 | 0.26 | 0.00 | 1.96% |
| 4-2 | 41.39 | 3.79 | 8.53 | 0.11 |  |  |  |  |  |  |  |  |  |
| 4-3 | 39.42 | 3.75 | 8.96 | 0.11 |  |  |  |  |  |  |  |  |  |
| 5-1 | 96.71 | 12.74 | 24.29 | 0.17 | 96.36 | 12.88 | 23.98 | 0.17 | 0.61 | 0.24 | 0.53 | 0.00 | 1.18% |
| 5-2 | 95.66 | 13.16 | 23.37 | 0.17 |  |  |  |  |  |  |  |  |  |
| 5-3 | 96.71 | 12.74 | 24.29 | 0.17 |  |  |  |  |  |  |  |  |  |
| 6-1 | 17.48 | 5.86 | 9.12 | 0.00 | 17.48 | 5.86 | 9.12 | 0.00 | 0.00 | 0.00 | 0.00 | 0.00 | 0.00% |
| 6-2 | 17.48 | 5.86 | 9.12 | 0.00 |  |  |  |  |  |  |  |  |  |
| 6-3 | 17.48 | 5.86 | 9.12 | 0.00 |  |  |  |  |  |  |  |  |  |
| 7-1 | 21.39 | 2.15 | 9.80 | 0.01 | 21.43 | 2.27 | 9.71 | 0.01 | 0.06 | 0.11 | 0.10 | 0.00 | 1.53% |
| 7-2 | 21.50 | 2.36 | 9.61 | 0.01 |  |  |  |  |  |  |  |  |  |
| 7-3 | 21.39 | 2.31 | 9.73 | 0.01 |  |  |  |  |  |  |  |  |  |
| 8-1 | 79.24 | 0.00 | 11.80 | 0.34 | 79.24 | 0.00 | 11.80 | 0.34 | 0.00 | 0.00 | 0.00 | 0.00 | 0.00% |
| 8-2 | 79.24 | 0.00 | 11.80 | 0.34 |  |  |  |  |  |  |  |  |  |
| 8-3 | 79.24 | 0.00 | 11.80 | 0.34 |  |  |  |  |  |  |  |  |  |
| 9-1 | 5.63 | 1.94 | 6.80 | 0.00 | 5.86 | 1.89 | 6.71 | 0.00 | 0.20 | 0.04 | 0.08 | 0.00 | 1.67% |
| 9-2 | 5.97 | 1.87 | 6.66 | 0.00 |  |  |  |  |  |  |  |  |  |
| 9-3 | 5.97 | 1.87 | 6.66 | 0.00 |  |  |  |  |  |  |  |  |  |
| 10-1 | 83.50 | 5.63 | 18.20 | 0.22 | 83.42 | 5.64 | 17.75 | 0.22 | 0.92 | 0.17 | 0.46 | 0.00 | 1.68% |
| 10-2 | 84.30 | 5.82 | 17.76 | 0.22 |  |  |  |  |  |  |  |  |  |
| 10-3 | 82.47 | 5.48 | 17.28 | 0.22 |  |  |  |  |  |  |  |  |  |

The collected color data were input into the model for prediction. The average pigment prediction values and their standard deviations were calculated. The RSD values for the pigment predictions were then computed and averaged to obtain the overall RSD. The results are shown in Table 3S. The RSD values were all below 2%, indicating that the model's prediction performance is stable.

**Table 4S**

Colour values of placebo granules.

| No. | B | G | R | B mean | G mean | R mean | B std | G std | R std | RSD |
| --- | --- | --- | --- | --- | --- | --- | --- | --- | --- | --- |
| 1-1 | 165 | 199 | 217 | 164 | 198 | 216 | 0.58 | 0.58 | 1.00 | 0.37% |
| 1-2 | 164 | 198 | 215 |  |  |  |  |  |  |  |
| 1-3 | 164 | 198 | 216 |  |  |  |  |  |  |  |
| 2-1 | 95 | 134 | 156 | 95 | 134 | 156 | 0.58 | 0.00 | 0.00 | 0.20% |
| 2-2 | 95 | 134 | 156 |  |  |  |  |  |  |  |
| 2-3 | 94 | 134 | 156 |  |  |  |  |  |  |  |
| 3-1 | 74 | 106 | 125 | 74 | 107 | 125 | 0.58 | 0.58 | 0.58 | 0.59% |
| 3-2 | 74 | 107 | 125 |  |  |  |  |  |  |  |
| 3-3 | 75 | 107 | 126 |  |  |  |  |  |  |  |
| 4-1 | 162 | 195 | 212 | 162 | 195 | 212 | 0.58 | 0.00 | 0.58 | 0.21% |
| 4-2 | 162 | 195 | 212 |  |  |  |  |  |  |  |
| 4-3 | 161 | 195 | 211 |  |  |  |  |  |  |  |
| 5-1 | 103 | 148 | 172 | 103 | 148 | 172 | 0.00 | 0.58 | 0.58 | 0.24% |
| 5-2 | 103 | 148 | 172 |  |  |  |  |  |  |  |
| 5-3 | 103 | 149 | 173 |  |  |  |  |  |  |  |
| 6-1 | 153 | 203 | 227 | 154 | 205 | 229 | 1.15 | 1.73 | 1.53 | 0.75% |
| 6-2 | 155 | 206 | 229 |  |  |  |  |  |  |  |
| 6-3 | 155 | 206 | 230 |  |  |  |  |  |  |  |
| 7-1 | 176 | 205 | 226 | 176 | 205 | 226 | 1.00 | 0.58 | 0.58 | 0.37% |
| 7-2 | 177 | 205 | 226 |  |  |  |  |  |  |  |
| 7-3 | 175 | 204 | 225 |  |  |  |  |  |  |  |
| 8-1 | 148 | 169 | 183 | 148 | 169 | 183 | 0.58 | 0.00 | 0.00 | 0.13% |
| 8-2 | 149 | 169 | 183 |  |  |  |  |  |  |  |
| 8-3 | 148 | 169 | 183 |  |  |  |  |  |  |  |
| 9-1 | 200 | 227 | 246 | 199 | 227 | 246 | 1.00 | 0.00 | 0.58 | 0.25% |
| 9-2 | 198 | 227 | 245 |  |  |  |  |  |  |  |
| 9-3 | 199 | 227 | 246 |  |  |  |  |  |  |  |
| 10-1 | 119 | 156 | 176 | 119 | 156 | 176 | 0.58 | 0.00 | 0.00 | 0.16% |
| 10-2 | 119 | 156 | 176 |  |  |  |  |  |  |  |
| 10-3 | 120 | 156 | 176 |  |  |  |  |  |  |  |

The placebo was prepared based on the average predicted pigment values. Three parallel image captures were repeated, and the average RGB values were calculated along with their standard deviations. The RSD values for the RGB values were then computed and averaged to obtain the overall RSD. The results are shown in Table 4S. The RSD values were all below 2%, indicating that the prepared placebo samples were uniform and stable.


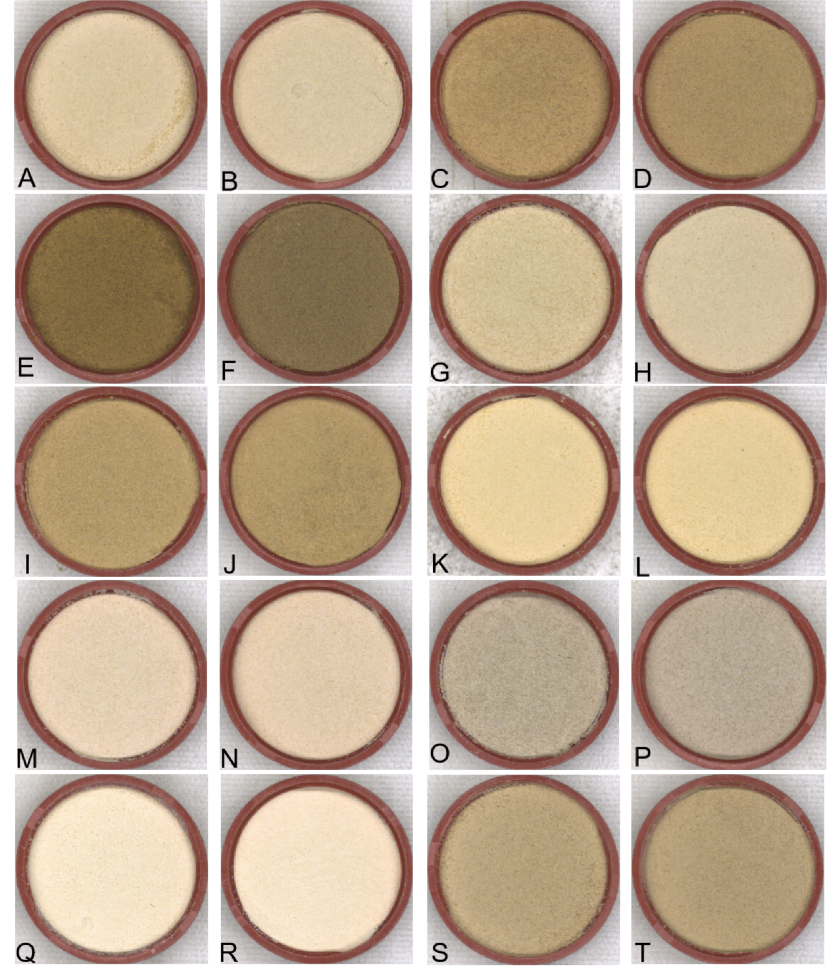


**Fig. 1S.** TCM granules and theirs placebo granules: (A) No.1 TCM granules, (B) No.1 placebo granules, (C) No.2 TCM granules, (D) No.2 placebo granules, (E) No.3 TCM granules, (F) No.3 placebo granules, (G) No.4 TCM granules, (H) No.4 placebo granules, (I) No.5 TCM granules, (J) No.5 placebo granules, (K) No.6 TCM granules, (L) No.6 placebo granules, (M) No.7 TCM granules, (N) No.7 placebo granules, (O) No.8 TCM granules, (P) No.8 placebo granules, (Q) No.9 TCM granules, (R) No.9 placebo granules, (S) No.10 TCM granules, (T) No.10 placebo granules.

**Table 5S**

Color data and similarity of TCM granules and their placebo.

| Name | B | G | R | L | a | b | ΔE | COS |
| --- | --- | --- | --- | --- | --- | --- | --- | --- |
| No.1 TCM granules | 161 | 198 | 218 | 81 | 1 | 21 | 2.0270 | 0.9999 |
| No.1 placebo | 164 | 198 | 216 | 81 | 1 | 19 |  |  |
| No.2 TCM granules | 98 | 142 | 170 | 61 | 4 | 27 | 4.9898 | 0.9998 |
| No.2 placebo | 95 | 134 | 156 | 57 | 3 | 24 |  |  |
| No.3 TCM granules | 63 | 103 | 126 | 45 | 3 | 26 | 5.1097 | 0.9983 |
| No.3 placebo | 74 | 107 | 125 | 46 | 3 | 21 |  |  |
| No.4 TCM granules | 152 | 191 | 209 | 78 | 0 | 22 | 3.2325 | 0.9998 |
| No.4 placebo | 162 | 195 | 212 | 79 | 1 | 19 |  |  |
| No.5 TCM granules | 107 | 153 | 178 | 64 | 3 | 28 | 2.0586 | 1.0000 |
| No.5 placebo | 103 | 148 | 172 | 62 | 2 | 27 |  |  |
| No.6 TCM granules | 161 | 210 | 233 | 85 | 1 | 27 | 1.6640 | 1.0000 |
| No.6 placebo | 154 | 205 | 229 | 84 | 1 | 28 |  |  |
| No.7 TCM granules | 180 | 208 | 229 | 85 | 3 | 17 | 2.0000 | 1.0000 |
| No.7 placebo | 176 | 205 | 226 | 83 | 3 | 17 |  |  |
| No.8 TCM granules | 144 | 168 | 181 | 69 | 1 | 14 | 1.9414 | 1.0000 |
| No.8 placebo | 148 | 169 | 183 | 70 | 2 | 13 |  |  |
| No.9 TCM granules | 202 | 230 | 246 | 92 | 1 | 16 | 1.4142 | 1.0000 |
| No.9 placebo | 199 | 227 | 246 | 91 | 2 | 16 |  |  |
| No.10 TCM granules | 125 | 162 | 183 | 68 | 2 | 22 | 3.0000 | 1.0000 |
| No.10 placebo | 119 | 156 | 176 | 65 | 2 | 22 |  |  |

The visual comparison between the Chinese herbal granules and the placebo is shown in Fig. 1S.The color difference between the Chinese herbal granules and their corresponding placebos was compared, and the ΔE and cos values were calculated. The results are shown in Table 5S. The average color difference (ΔE) was 2.7437, and the average cos value was 0.9998. This indicates that the model's fit is as expected and performs well on unseen data, which can indirectly suggest that the model has not overfitted.
