# Supplementary material for: Revolutionizing Chinese medicine granule placebo with a machine learning four-color model
Source: Chin Med. 2025 Apr 1;20:43. doi: 10.1186/s13020-024-01055-0 (PMC11963323; doi:10.1186/s13020-024-01055-0)
Supplement: Supplementary file 2 — Supplementary material 2. [file 13020_2024_1055_MOESM2_ESM.docx]

**Appendix 1**

Information on the 52 kinds of TCM granules.

| TCM Granules | Manufacture company | Batch |
| --- | --- | --- |
| Xuanfei Baidu Granules | Shandong Buchang Pharmaceutical Co., Ltd. | 210209 |
| Xiao'er Yanbian Granules | Chengde Yanfeng Pharmaceutical Co., Ltd. | 23823004 |
| Wushicha granules | Huangshi Yanwu Pharmaceutical Co., Ltd. | 20230402 |
| Xiqingguo Granules | Guangxi Jimin Pharmaceutical Co., Ltd. | 230210 |
| Shenqi Jianwei Granules | Jiangsu Zhongxing Pharmaceutical Co., Ltd. | 230102 |
| Sanjiu Weitai Granules | China Resources Sanjiu Pharmaceutical Co., Ltd. | 2210035F |
| Pipa Zhike Granules | Anhui Zhanghengchun Pharmaceutical Co., Ltd. | 2304013 |
| Fufang Guazijin Granules | Wangshi Pharmaceuticals | 20220929 |
| Huoxiang Zhengqi Granules | Sichuan Fengchun Pharmaceutical Co., Ltd. | 230131 |
| Yinhuang granules | Hebei Guojin Pharmaceutical Co., Ltd. | B2303271 |
| Xiaochaihu Granules | Sichuan Fengchun Pharmaceutical Co., Ltd. | 230341 |
| Fuyang Granules | Chengdu Dikang Pharmaceutical Co., Ltd. | 211117 |
| Banlangen granules | Sichuan Yike Pharmaceutical Co., Ltd. | 230402 |
| Xiao'er Chaigui antipyretic granules | Sunflower Pharmaceutical | 230112 |
| Ganmao Qingre granules | Hebei Wansui Pharmaceutical Co., Ltd. | 221277 |
| Yinqiao Jiedu Granules | Guangxi Shengmin Pharmaceutical Co., Ltd. | 230302 |
| Xiasangju Granules | Sichuan Yike Pharmaceutical Co., Ltd. | 230211 |
| Feining Granules | Jilin Yimintang Pharmaceutical Co., Ltd. | 230341 |
| Fufang Luohanguo Zhike Granules | Guangxi Guigang Xiashan Pharmaceutical Factory | 230101 |
| Tongqiao Biyan Granules | Chengdu Dikang Pharmaceutical Co., Ltd. | 220942 |
| Xiao'er Feire Kechuan Granules | Sunflower Pharmaceutical | 22120181 |
| Xiao'er Qixingcha Granules | Guangxi Shengmin Pharmaceutical Co., Ltd. | 230302 |
| Yiqing granules | Shanxi Aoer Pharmaceutical Co., Ltd. | 230309 |
| Fufang Jinyinhua Granules | Hebei Guojin Pharmaceutical Co., Ltd. | C2302111 |
| Weisu Granules | Yangtze River Pharmaceutical Group |  |
| Qizhi Weitong Granules | Liaoning Huarun Benxi Three Medicines Co., Ltd. | 20191241 |
| Baohe Granules | Anhui Jiufang Pharmaceutical Co., Ltd. | 230404 |
| Xiao'er Jiegan Granules | Shandong Mingren Furuida Pharmaceutical Co., Ltd. | 23105013 |
| Jinji Granules | Zhongshan Hengsheng Pharmaceutical Co., Ltd. | 20230401 |
| Jieyu Anshen Granules | Jilin Jinghui Pharmaceutical Co., Ltd. | 230405 |
| Juhong granules | Jiangxi Minkang Pharmaceutical Co., Ltd. | 230247 |
| Dahuang Tongbian Granules | Jiangsu Chenpai Pharmaceutical Group Co., Ltd. | 2304251 |
| Fufang Xianshihu Granules | Wenzhou Haihe Pharmaceutical Co., Ltd. | 20221201 |
| Shenling Baizhu Granules | Shanxi Huakang Pharmaceutical Co., Ltd. | 20230401 |
| Xiangsha Pingwei Granules | Yunnan Tengyao Pharmaceutical Co., Ltd. | 20230476 |
| Jianwei Xiaoyan Granules | Inner Mongolia Datang Pharmaceutical Co., Ltd. | 22648038 |
| Kunzhong Fei'er Chanji Granules | Kunming Traditional Chinese Medicine Factory Co., Ltd. | 520894 |
| Xiaoqinglong Granules | Sichuan Taile Pharmaceutical Co., Ltd. | 230306 |
| Changyanning Granules | Hulu Aristocratic Family | 230101 |
| Qinghouyan granules | Guangxi Wanshoutang Pharmaceutical Co., Ltd. | 230405 |
| Renshen Wuweizi granules | Jilin Hongjing Pharmaceutical Co., Ltd. | 20221101 |
| Juhong Tanke Cough Granules | Huluwa Pharmaceutical | 230212 |
| Jinxuan Liyan Granules | Hangzhou Huadong Pharmaceutical Group Kangrun Pharmaceutical Co., Ltd. | 230301 |
| Honghua Xiaoyao Granules | Jiangxi Juwang Pharmaceutical Co., Ltd. | 230203/5 |
| Yupingfeng Granules | China National Pharmaceutical Group Guangdong Global Pharmaceutical Co., Ltd. | 230408 |
| Fuke Tiaojing Granules | Li Shizhen Pharmaceutical Group Co., Ltd. | 202211004 |
| Xiao'er Changweikang Granules | Wenzhou Haihe Pharmaceutical Co., Ltd. | 230306 |
| Qibao Meiran Granules | Kunming Traditional Chinese Medicine Factory Co., Ltd. | 531029 |
| Nuanwei Shule Granules | Kunming Traditional Chinese Medicine Factory Co., Ltd. | 522584 |
| Jianpi Shengxue Granules | Jianmin Pharmaceutical Group Co., Ltd. | 230440 |
| Qingxuan Zhike Granules | Suzhong Pharmaceutical Group Co., Ltd. | 22112103 |
| Sijunzi Granules | Li Shizhen Pharmaceutical Group Co., Ltd. | 202305001 |
| Xuanfei Baidu Granules | Shandong Buchang Pharmaceutical Co., Ltd. | 210209 |
| Xiao'er Yanbian Granules | Chengde Yanfeng Pharmaceutical Co., Ltd. | 23823004 |
| Wushicha granules | Huangshi Yanwu Pharmaceutical Co., Ltd. | 20230402 |
| Xiqingguo Granules | Guangxi Jimin Pharmaceutical Co., Ltd. | 230210 |
| Shenqi Jianwei Granules | Jiangsu Zhongxing Pharmaceutical Co., Ltd. | 230102 |
| Sanjiu Weitai Granules | China Resources Sanjiu Pharmaceutical Co., Ltd. | 2210035F |
| Pipa Zhike Granules | Anhui Zhanghengchun Pharmaceutical Co., Ltd. | 2304013 |
| Fufang Guazijin Granules | Wangshi Pharmaceuticals | 20220929 |
| Huoxiang Zhengqi Granules | Sichuan Fengchun Pharmaceutical Co., Ltd. | 230131 |
| Yinhuang granules | Hebei Guojin Pharmaceutical Co., Ltd. | B2303271 |
| Xiaochaihu Granules | Sichuan Fengchun Pharmaceutical Co., Ltd. | 230341 |
| Fuyang Granules | Chengdu Dikang Pharmaceutical Co., Ltd. | 211117 |
| Banlangen granules | Sichuan Yike Pharmaceutical Co., Ltd. | 230402 |
| Xiao'er Chaigui antipyretic granules | Sunflower Pharmaceutical | 230112 |
| Ganmao Qingre granules | Hebei Wansui Pharmaceutical Co., Ltd. | 221277 |
| Yinqiao Jiedu Granules | Guangxi Shengmin Pharmaceutical Co., Ltd. | 230302 |
| Xiasangju Granules | Sichuan Yike Pharmaceutical Co., Ltd. | 230211 |
| Feining Granules | Jilin Yimintang Pharmaceutical Co., Ltd. | 230341 |
| Fufang Luohanguo Zhike Granules | Guangxi Guigang Xiashan Pharmaceutical Factory | 230101 |
| Tongqiao Biyan Granules | Chengdu Dikang Pharmaceutical Co., Ltd. | 220942 |
| Xiao'er Feire Kechuan Granules | Sunflower Pharmaceutical | 22120181 |
| Xiao'er Qixingcha Granules | Guangxi Shengmin Pharmaceutical Co., Ltd. | 230302 |
| Yiqing granules | Shanxi Aoer Pharmaceutical Co., Ltd. | 230309 |
| Fufang Jinyinhua Granules | Hebei Guojin Pharmaceutical Co., Ltd. | C2302111 |
| Weisu Granules | Yangtze River Pharmaceutical Group |  |
| Qizhi Weitong Granules | Liaoning Huarun Benxi Three Medicines Co., Ltd. | 20191241 |
| Baohe Granules | Anhui Jiufang Pharmaceutical Co., Ltd. | 230404 |
| Xiao'er Jiegan Granules | Shandong Mingren Furuida Pharmaceutical Co., Ltd. | 23105013 |
| Jinji Granules | Zhongshan Hengsheng Pharmaceutical Co., Ltd. | 20230401 |
| Jieyu Anshen Granules | Jilin Jinghui Pharmaceutical Co., Ltd. | 230405 |
| Juhong granules | Jiangxi Minkang Pharmaceutical Co., Ltd. | 230247 |
| Dahuang Tongbian Granules | Jiangsu Chenpai Pharmaceutical Group Co., Ltd. | 2304251 |
| Fufang Xianshihu Granules | Wenzhou Haihe Pharmaceutical Co., Ltd. | 20221201 |
| Shenling Baizhu Granules | Shanxi Huakang Pharmaceutical Co., Ltd. | 20230401 |
| Xiangsha Pingwei Granules | Yunnan Tengyao Pharmaceutical Co., Ltd. | 20230476 |
| Jianwei Xiaoyan Granules | Inner Mongolia Datang Pharmaceutical Co., Ltd. | 22648038 |
| Fei'er Ganji Granules | Kunming Traditional Chinese Medicine Factory Co., Ltd. | 520894 |
| Xiaoqinglong Granules | Sichuan Taile Pharmaceutical Co., Ltd. | 230306 |
| Changyanning Granules | Hulu Aristocratic Family | 230101 |
| Qinghouyan granules | Guangxi Wanshoutang Pharmaceutical Co., Ltd. | 230405 |
| Renshen Wuweizi granules | Jilin Hongjing Pharmaceutical Co., Ltd. | 20221101 |
| Juhong Tanke Cough Granules | Huluwa Pharmaceutical | 230212 |
| Jinxuan Liyan Granules | Hangzhou Huadong Pharmaceutical Group Kangrun Pharmaceutical Co., Ltd. | 230301 |
| Honghua Xiaoyao Granules | Jiangxi Juwang Pharmaceutical Co., Ltd. | 230203/5 |
| Yupingfeng Granules | China National Pharmaceutical Group Guangdong Global Pharmaceutical Co., Ltd. | 230408 |
| Fuke Tiaojing Granules | Li Shizhen Pharmaceutical Group Co., Ltd. | 202211004 |
| Xiao'er Changweikang Granules | Wenzhou Haihe Pharmaceutical Co., Ltd. | 230306 |
| Qibao Meiran Granules | Kunming Traditional Chinese Medicine Factory Co., Ltd. | 531029 |
| Nuanwei Shule Granules | Kunming Traditional Chinese Medicine Factory Co., Ltd. | 522584 |
| Jianpi Shengxue Granules | Jianmin Pharmaceutical Group Co., Ltd. | 230440 |
| Qingxuan Zhike Granules | Suzhong Pharmaceutical Group Co., Ltd. | 22112103 |
| Sijunzi Granules | Li Shizhen Pharmaceutical Group Co., Ltd. | 202305001 |
