# Supplementary material for: Revolutionizing Chinese medicine granule placebo with a machine learning four-color model
Source: Chin Med. 2025 Apr 1;20:43. doi: 10.1186/s13020-024-01055-0 (PMC11963323; doi:10.1186/s13020-024-01055-0)
Supplement: Supplementary file 3 — Supplementary material 3. [file 13020_2024_1055_MOESM3_ESM.docx]

**Appendix 2**

Information on simulated granular excipients.

| Excipients | Manufacture company |
| --- | --- |
| Lemon yellow | Shanghai Dyestuffs Research Institute Co., Ltd. |
| Carmine | Shanghai Dyestuffs Research Institute Co., Ltd. |
| Indigo | Shanghai Dyestuffs Research Institute Co., Ltd. |
| Caramel color | Shanghai Aipu Food Industry Co., Ltd. |
| Lactose | Zhenjiang Kangfu Bioengineering Co., Ltd. |
| Dextrin | Anhui Shanhe Pharmaceutical Accessories Co., Ltd. |
